# Human Papillomavirus-16 Infection in Advanced Oral Cavity Cancer Patients Is Related to an Increased Risk of Distant Metastases and Poor Survival

**DOI:** 10.1371/journal.pone.0040767

**Published:** 2012-07-12

**Authors:** Li-Ang Lee, Chung-Guei Huang, Chun-Ta Liao, Li-Yu Lee, Chuen Hsueh, Tse-Ching Chen, Chien-Yu Lin, Kang-Hsing Fan, Hung-Ming Wang, Shiang-Fu Huang, I-How Chen, Chung-Jan Kang, Shu-Hang Ng, Shu-Li Yang, Kuo-Chien Tsao, Yu-Liang Chang, Tzu-Chen Yen

**Affiliations:** 1 Head and Neck Oncology Group, Chang Gung Memorial Hospital and Chang Gung University, Taoyuan, Taiwan; 2 Department of Otolaryngology-Head and Neck Surgery, Chang Gung Memorial Hospital and Chang Gung University, Taoyuan, Taiwan; 3 Department of Pathology, Chang Gung Memorial Hospital and Chang Gung University, Taoyuan, Taiwan; 4 Department of Radiation Oncology, Chang Gung Memorial Hospital and Chang Gung University, Taoyuan, Taiwan; 5 Medical Oncology, Chang Gung Memorial Hospital and Chang Gung University, Taoyuan, Taiwan; 6 Department of Diagnostic Radiology, Chang Gung Memorial Hospital and Chang Gung University, Taoyuan, Taiwan; 7 Department of Laboratory Medicine, Chang Gung Memorial Hospital and Chang Gung University, Taoyuan, Taiwan; 8 Department of Oral and Maxillofacial Surgery, Chang Gung Memorial Hospital and Chang Gung University, Taoyuan, Taiwan; 9 Department of Nuclear Medicine and Molecular Imaging Center, Chang Gung Memorial Hospital and Chang Gung University, Taoyuan, Taiwan; IPO, Inst Port Oncology, Portugal

## Abstract

**Background:**

Human papillomavirus (HPV) is an oncogenic virus causing oropharyngeal cancers and resulting in a favorable outcome after the treatment. The role of HPV in oral cavity squamous cell carcinoma (OSCC) remains ambiguous.

**Objective:**

This study aimed to examine the effect of HPV infection on disease control among patients with OSCC following radical surgery with radiation-based adjuvant therapy.

**Patients and Method:**

We prospectively followed 173 patients with advanced OSCC (96% were stage III/IV) who had undergone radical surgery and adjuvant therapy between 2004 and 2006. They were followed between surgery and death or up to 60 months. Surgical specimens were examined using a PCR-based HPV blot test. The primary endpoints were the risk of relapse and the time to relapse; the secondary endpoints were disease-free survival, disease-specific survival, and overall survival.

**Results:**

The prevalence of HPV-positive OSCC was 22%; HPV-16 (9%) and HPV-18 (7%) were the genotypes most commonly encountered. Solitary HPV-16 infection was a poor predictor of 5-year distant metastases (hazard ratio, 3.4; 95% confidence interval, 1.4–8.0; *P* = 0.005), disease-free survival (*P* = 0.037), disease-specific survival (*P* = 0.006), and overall survival (*P* = 0.010), whereas HPV-18 infection had no impact on 5-year outcomes. The rate of 5-year distant metastases was significantly higher in the HPV-16 or level IV/V metastasis group compared with both the extracapsular spread or tumor depth ≥11-mm group and patients without risk factors (*P*<0.001).

**Conclusions:**

HPV infections in advanced OSCC patients are not uncommon and clinically relevant. Compared with HPV-16-negative advanced OSCC patients, those with a single HPV-16 infection are at higher risk of distant metastases and poor survival despite undergoing radiation-based adjuvant therapy and require a more aggressive adjuvant treatment and a more thorough follow-up.

## Introduction

Human papillomavirus (HPV) is a well-known oncogenic virus often observed in patients who have had a favorable outcome after the treatment of oropharyngeal cancers [1]–[11]. The causative mechanism is unclear but may be partially related to the radiosensitivity of the primary tumor or the less aggressive nature of tumors that are small at presentation (i.e., primary tumor [T]: T1–T2) [1], [8], [9], [11]. HPV-positive tumors usually coincide with more regional lymph node (N) metastases (i.e., N2–N3) [4], [7]–[9], [11], though some patients with HPV-positive tumors show fewer nodal metastases [6]. Analyses of failure patterns are important for the post-treatment surveillance of early disease recurrence because the only chance for survival in patients with recurrent tumors is the early detection of lesions that can serve as the targets of salvage therapy. The majority of previous studies have focused on the correlations between HPV infection and various measures of survival; few studies have addressed the failure patterns at local, regional, and distant sites [8], [11].

However, the role of HPV in oral cavity squamous cell carcinoma (OSCC) remains ambiguous because of the relatively small number of recorded OSCC patients in comparison with the larger population of oropharyngeal cancer [3], [6], [9], [10], [12]–[16]. In southern Asia, OSCC is an endemic cancer with an etiology that is distinct from that seen in the United States and Europe. Generally, OSCC patients with resectable tumors, but without distant metastasis, undergo radical surgery as the primary treatment in southern Asia. In the case of advanced OSCC (T4 lesion, lymph node metastasis, margin status of ≤4 mm] or extracapsular spread [ECS]), postoperative radiotherapy (RT) or concomitant chemoradiation therapy (CCRT) is used for adjuvant therapy [17], [18].

Several questions about the role of HPV in advanced OSCC patients who require adjuvant therapy following radical surgery in southern Asia remain unanswered. For example, what is the incidence of HPV infections among OSCC patients? Are the clinical and biological behaviors of HPV in OSCC the same as those in oropharyngeal SCC? Are different treatment strategies and follow-up protocols appropriate in HPV-positive OSCC patients? Does HPV infection affect the outcomes of postoperative adjuvant therapy? To answer these questions, we studied a large cohort of patients with previously untreated OSCC who underwent radical surgery with or without adjuvant therapy; in particular, we focused on the impact of HPV infections on the outcomes of radiation-based adjuvant therapy for advanced OSCC. Accordingly, this study aimed to test the hypothesis that HPV infections among advanced OSCC patients are associated with a decreased risk of disease relapse, including local recurrence, neck recurrence, and distant metastasis, and therefore improve the rates of survival, including disease-free survival (DFS), disease-specific survival (DSS), and overall survival (OS).

## Materials and Methods

### Patients

The Institutional Review Board at Chang Gung Memorial Hospital approved this study, which complied with the Declaration of Helsinki. All participants provided written informed consent. The inclusion criteria were as follows: a histological diagnosis of OSCC, the presence of a previously untreated tumor scheduled for radical surgery with neck dissection (ND), the absence of other suspected distant metastatic lesions detected by imaging, and a willingness to undergo imaging-guided biopsy or exploratory surgery if necessary. The exclusion criteria included a refusal or inability to undergo radical surgery.

Between 2004 and 2006, 333 patients were prospectively included in this study. All patients consented to and participated in the long-term outcome survey program of the Head and Neck Oncology Group at the Chang Gung Memorial Hospital. All participants underwent an extensive presurgical evaluation that included a medical history and a complete physical examination, flexible fiberoptic pharyngoscopy, a complete blood count, routine blood biochemistry, CT or MRI scans of the head and neck, chest radiographs, bone scans, and liver ultrasonography. Cancer staging was performed according to the 2002 American Joint Committee on Cancer 6th edition staging criteria [19].

All patients underwent radical excision of the primary tumor with ≥1 cm gross safety margins (both peripheral and deep margins). Classic radical or modified NDs (level I–V) were performed in the patients with clinically positive lymph node disease. Supra-omohyoid NDs (level I–III) were performed in clinically node-negative patients. Most of the uncomplicated patients underwent surgery alone except for those who unexpectedly had close margins ≤4 mm and/or positive lymph nodes as identified by pathological examinations. In this study, the subjects who underwent adjuvant therapy were considered as advanced OSCC patients. The indications for postoperative RT (60–66 Gy) included pathological T4 tumor, a positive lymph node, or a close margin ≤4 mm. ECS or multiple lymph node metastases were the reasons for the administration of CCRT with 50 mg/m^2^ cisplatin biweekly plus 800 mg daily oral tegafur and 60 mg leucovorin, or 30 mg/m^2^ weekly cisplatin [17], [18]. In the present study, 173 (52%) of the 333 OSCC patients underwent radical surgery followed by adjuvant therapy for advanced OSCC for the reasons stated above.

### Clinicopathologic Characteristics

Patient data were extracted from medical records and classified according to our previously identified risk factors for OSCC, which were described in detail elsewhere [20]. The clinical and pathologic characteristics of interest included sex, age of disease onset, alcohol drinking, betel quid chewing, cigarette smoking, tumor subsite, differentiation, pathological T-status, pathological N-status, pathological stage, ECS, level IV/V metastases, treatment mode, and patient status at the last follow-up. Tumor subsite was determined by direct oral inspection and confirmed by pathological examination. Local recurrence was defined as a positive biopsy in the area of the primary tumor after a radical surgery as determined by a negative post-treatment screen. A neck recurrence was defined as a positive cytology/biopsy in the cervical lymphatic region after primary surgery. An incident distant metastasis was identified through biopsy or by imaging, as verified by our tumor board.

### HPV Detection

Excised tumor samples were collected during radical surgery. DNA was extracted from paraffin-embedded tumor samples using a Lab Turbo 48 automatic nucleic acid extraction system and a Lab Turbo Virus Mini Kit LVN500 (Taigen, Taipei, Taiwan). Finally, 50 µL of DNA solution was eluted, and 1 µL was used as the PCR template. HPV infection was diagnosed in subjects using PCR on the HPV L1 gene. HPV DNA was amplified with MY11/biotinylated GP6+ primers, which targeted the L1 region and produced a 192-bp DNA fragment. The PCR reaction volume was 25 µL, which included a 2-µL aliquot of purified DNA. In the positive cases, the HPV L1 gene was genotyped using an HPV Blot kit (EasyChip™, King Car Ltd., Yilan, Taiwan) that can differentiate the 39 HPV types (HPV 6, 11, 16, 18, 26, 31, 32, 33, 35, 37, 39, 42, 43, 44, 45, 51, 52, 53, 54, 55, 56, 58, 59, 61, 62, 66, 67, 68, 69, 70, 72, 74, 82, CP8061, CP8304, L1AE5, MM4, MM7, and MM8). HPV type-specific probes were immobilized on a nylon membrane, which was used for reverse blot hybridization to detect HPV DNA in a single assay. The HPV types were determined using a visual assessment protocol provided by the manufacturer [21]–[23].

### Study Endpoints

The primary endpoint was time to disease relapse including local recurrence, neck recurrence, and distant metastasis. The secondary endpoints were DFS, DSS, and OS. DFS was calculated as the date of primary surgery to the date of disease relapse. DSS was calculated as the date of primary surgery to the date of death caused by a disease recurrence, and OS was defined as the time period between primary surgery and death caused by any reason.

### Statistical Analysis

Follow-up visits continued until December 2011. All patients received follow-up examinations for at least 60 months after surgery or until death. The procedure used for selecting the optimal cutoff values for clinicopathological factors has been previously described (20). Five-year local control, neck control, distant metastasis, DFS, DSS, and OS rates were computed using the Kaplan-Meier method (log-rank test). Univariate and multivariate analyses were used to identify independent predictors of 5-year outcomes. Independent prognostic factors were identified using multivariate Cox regression analysis with a forward selection procedure. Statistical analyses were performed using the SPSS software (version 17.0; SPSS Inc., Chicago, IL, USA). A two-sided P value <0.05 was considered statistically significant.

## Results

### Characterization of Patients

During the study period, we recruited 333 OSCC patients (316 males and 17 females; mean age at onset, 51 years; age range, 25–83 years). A total of 240 patients (72%) reported drinking alcohol, 284 (85%) reported chewing betel quid, and 290 (87%) reported smoking cigarettes. Nineteen patients (6%) underwent primary tumor excision only, and the other 314 (94%) underwent ND in addition to primary tumor excision. The results of the pathological staging were as follows: pT1 (15%); pT2 (41%); pT3 (15%); pT4 (29%); pNx (6%); pN0 (55%); pN1 (12%); pN2b (24%); and pN2c (3%). When pNx (in the absence of ND) was classified as pN0, the pathological stages were as follows: p-stage I (14%), p-stage II (29%), p-stage III (15%), and p-stage IV (43%). The 5-year control and survival rates for all of the OSCC patients were as follows: local control, 89%; neck control, 86%; distant metastases, 12%; DFS, 73%; DSS, 79%; and OS, 66%.

Among the 333 patients, 52% had advanced OSCC and underwent radical surgery followed by adjuvant therapy (RT, n = 81; CCRT, n = 92), whereas 48% were the uncomplicated OSCC patients who underwent surgery alone. The tumor aggressiveness that was observed in the advanced OSCC patients was distinctively different from that of the uncomplicated OSCC patients in terms of pathological T-status (pT3-4: 70% vs. 30%, P<0.001), pathological N-status (pN1-2: 72% vs. 4%, P<0.001), pathological stage (p-stage III-IV: 96% vs. 17%, P<0.001), and ECS (positive: 45% vs. 2%, P<0.001). The 5-year control/survival rates were significantly worse among the advanced OSCC patients compared with those of the uncomplicated OSCC patients: local control (84% vs. 93%, P = 0.003), neck control (81% vs. 92%, P = 0.007), distant metastases (22% vs. 2%, P<0.001), DFS (60% vs. 87%, P<0.001), DSS (65% vs. 93%, P<0.001), and OS (50% vs. 83%, P<0.001).

The median duration of follow-up for the advanced OSCC patients was 58 months (mean, 47 months; range, 2–95 months). At the time of the analysis, 81 of the 173 patients (47%) were alive, and 92 (53%) were dead (59 due to the primary cancer, 20 due to other cancers, and 13 due to non-cancer causes). Twenty-five patients (15%) developed local recurrences, 31 (18%) had neck recurrences, and 35 (20%) experienced distant metastases. A total of 47 patients (27%) exhibited local and/or neck recurrence, salvage therapy was performed in 29 individuals (62%) and 21 (72%) dead at the time of the analysis.

### HPV Status, Disease Relapse, and Survival

The overall prevalence of HPV infection among the OSCC patients was 21.3% (n = 71), and the 3 most common genotypes, including single and multiple infections, were as follows: HPV-16 (9.6%, n = 26), HPV-18 (7.8%, n = 23), and HPV-52 (2.4%, n = 6). The proportion of HPV-positive cases among the advanced OSCC patients was similar to that among the uncomplicated OSCC patients (22.0% vs. 20.6%, P = 0.765). The distributions of HPV-16 (9.2% vs. 6.3%), HPV-18 (6.9% vs. 6.9%), and HPV-52 (0.6% vs. 3.1%) were similar in both groups (all P>0.05).

**Figure 1:**
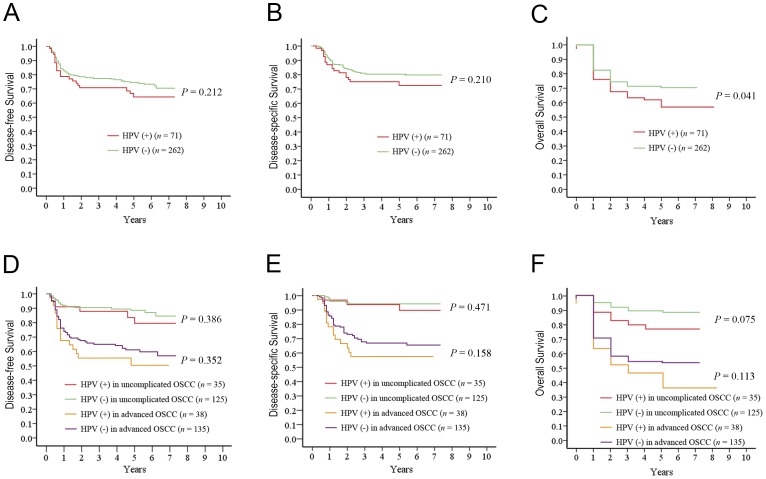
Five-year survivals by HPV status for patients with OSCC. A, DFS by HPV status for the entire population; B, DSS by HPV status for the entire population; C, OS by HPV status for the entire population; D, DFS by HPV status for uncomplicated and advanced OSCC patients; E, DSS by HPV status for uncomplicated and advanced OSCC patients; F, OS by HPV status for uncomplicated and advanced OSCC patients.

We calculated the 5-year DFS, DSS, and OS rates among the OSCC patients according to HPV status. Compared with the HPV-negative patients, those with HPV-positive tumors had similar rates of DFS (P = 0.212; Fig. 1A) and DSS (P = 0.210; Fig. 1B), but had a less favorable OS (P = 0.041; Fig. 1C) regardless of the treatment modality. When the entire cohort was further analyzed according to the need for treatment, the impact of HPV on 5-year DFS and DSS was not significant in both the uncomplicated and advanced groups (all P>0.05; Fig. 1D & Fig. 1E). Among the uncomplicated and advanced OSCC patients, the HPV-positive cases seemed to have a shorter time to death than those without detectable HPV, although these difference were not statistically significant (P = 0.075 & 0.112, respectively; Fig. 1F).

To shed more light on the influence of the HPV genotype on the study endpoints, we further classified the study participants according to different HPV statuses (i.e., solitary HPV-infection, solitary HPV-18 infection, HPV-16 and/or HPV-18 infection, high-risk HPV infection, and HPV infection). The 5-year outcomes were calculated in both the advanced OSCC group and in the uncomplicated OSCC group. There were no significant differences in time-to-recurrence or time-to-death among the HPV subgroups in the uncomplicated OSCC group (all P>0.05; data not shown). Table 1 shows that solitary HPV-16 infection was associated with a significantly higher rate of distant metastases (56% vs. 19%, P = 0.007), lower DSS (37% vs. 68%, P = 0.025) and lower OS (25% vs. 53%, P = 0.028) in advanced OSCC patients. HPV-16 and/or HPV-18 infection was significantly related to an increased rate of distant metastases (43% vs. 18%, P = 0.031). In contrast, solitary HPV-18 infection, high-risk HPV infection, and HPV infection (Fig. 1) did not show a statistically significant association with 5-year control and survival rates.

**Table 1:** HPV statuses and 5-year control and survival rates in advanced OSCC patients (*n* = 173).

Characteristics	Local control	*P*	Neck control	*P*	Distant metastases	*P*	Disease-free survival	*P*	Disease-specificsurvival	*P*	Overallsurvival	*P*
(*n*, %)	(5-yr %, *n* event)		(5-yr %, *n* event)		(5-yr %, *n* event)		(5-yr %, *n* event)		(5-yr %, *n* event)		(5-yr %, *n*event)	
Solitary HPV-16		0.774		0.726		0.007		0.058		0.025		0.028
No (157, 91)	84 (23)		82 (28)		19 (28)		62 (61)		68 (50)		53 (80)	
Yes (16, 9)	87 (2)		77 (3)		56 (7)		38 (9)		37 (9)		25 (12)	
Solitary HPV-18		0.797		0.416		0.685		0.955		0.996		0.602
No (161, 93)	86 (23)		81 (30)		20 (32)		60 (65)		66 (55)		46 (87)	
Yes (12, 7)	83 (2)		92 (1)		25 (3)		58 (5)		67 (4)		58 (5)	
HPV-16 and/or HPV18		0.378		0.634		0.031		0.125		0.057		0.088
No (143, 83)	86 (20)		81 (27)		18 (25)		62 (55)		69 (45)		49 (73)	
Yes (30, 17)	83 (5)		87 (4)		43 (10)		50 (15)		53 (14)		37 (19)	
High-risk HPV		0.361		0.653		0.147		0.188		0.078		0.073
No (137, 79)	86 (19)		81 (26)		18 (25)		61 (53)		69 (43)		50 (69)	
Yes (36, 21)	83 (6)		86 (5)		28 (10)		53 (17)		56 (16)		36 (23)	
HPV		0.512		0.528		0.230		0.352		0.158		0.113
No (135, 78)	86 (19)		81 (26)		19 (25)		61 (53)		68 (43)		50 (68)	
Yes (38, 22)	84 (6)		87 (5)		27 (10)		55 (17)		58 (16)		37 (24)	

HPV: human papillomavirus. OSCC: oral squamous cell carcinoma. *n*: patient number. W-M: Well-moderate.

**Figure 2:**
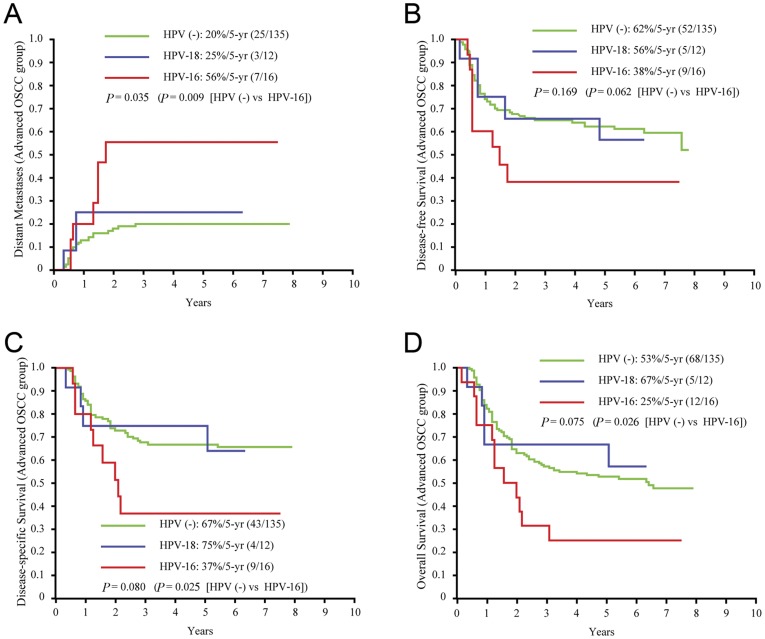
Five-year outcomes by HPV subgroups for patients with advanced OSCC. A, time to distant metastases; B, DFS; C, DSS; D, OS.

To further comparison, we further divided HPV infection into 3 subgroups according to genotype frequency (i.e., HPV-negative [n = 135], solitary HPV-16 infection [n = 16], and solitary HPV-18 infection [n = 12). Among the advanced OSCC patients, single HPV-16 infection was unrelated to local control or neck recurrence. Solitary HPV-16 infection was associated with a higher rate of distant metastases than the HPV-negative status (56% vs. 20%, P = 0.009; Fig. 2A). Despite postoperative adjuvant therapy, HPV-16 infection seemed to have a negative, although not statistically significant, impact on DFS compared with HPV-negative status (38% vs. 62%, P = 0.062; Fig. 2B). Moreover, advanced OSCC patients with HPV-16 infection had significantly lower DSS and OS rates than HPV-negative subjects (DSS: 37% vs. 67%, P = 0.025; OS: 25% vs. 53%, P = 0.026; Fig. 2C & Fig. 2D). Nevertheless, the differences in time-to-relapse and time-to-death between HPV-18 and HPV-negative cases were not statistically significant (Fig. 2).

Table 2 demonstrates the clinical/pathological characteristics associated with lack of HPV, HPV-16, and HPV-18 infections among the advanced OSCC patients. The patients who were HPV-positive (with either HPV-16 or HPV-18) had a higher rate of poor differentiation than those who were HPV-negative (P = 0.013). The HPV-18 group seemed to have a slightly lower rate of pT3-4 than both the HPV-negative and HPV-16 groups (P = 0.069). The HPV-16 group seemed to have a higher rate of distant metastases than the HPV-negative and HPV-18 groups (P = 0.064). The remaining endpoints (i.e., local recurrence, neck recurrence, relapse, secondary primary tumors, death, and cause of death) did not differ significantly among the study groups (all P>0.05).

**Table 2:** Clinicopathological characteristics of patients with advanced OSCC according to their HPV status.

Characteristic	All cases (*n* = 163)^a^	HPV (-) (*n* = 135)	Solitary HPV-16(*n* = 16)	Solitary HPV-18(n = 12)	*P*
	*n* (%)	*n* (%)	*n* (%)	*n* (%)	
Sex					0.707
Male	156 (96)	129 (96)	15 (94)	12 (100.0)	
Female	7 (4)	6 (4)	1 (6)	0	
Age of disease onset (years)					0.921
≤40	16 (10)	13 (10)	2 (13)	1 (8)	
>40	147 (90)	122 (90)	14 (87)	11 (92)	
Alcohol drinking					0.715
No	43 (26)	37 (27)	4 (25)	2 (17)	
Yes	120 (74)	98 (73)	12 (75)	10 (83)	
Betel quid chewing					0.669
No	21 (13)	18 (13)	1 (6)	2 (17)	
Yes	142 (87)	117 (87)	15 (94)	10 (83)	
Cigarette smoking					0.550
No	22 (14)	20 (15)	1 (6)	1 (8)	
Yes	141 (86)	115 (85)	15 (94)	11 (92)	
Tumor subsite					0.952
Tongue	42 (26)	34 (25)	5 (31)	3 (25)	
Mouth floor	7 (4)	6 (4)	1 (6)	0	
Lip	1 (1)	1 (1)	0	0	
Buccal	60 (37)	50 (37)	5 (31)	5 (42)	
Gum	35 (22)	28 (21)	5 (31)	2 (17)	
Hard palate	5 (3)	4 (3)	0	1 (8)	
Retromolar	13 (8)	12 (9)	0	1 (8)	
Differentiation					0.013
Well/moderate	150 (92)	128 (95)	13 (81)	9 (75)	
Poor	13 (8)	7 (5)	3 (19)	3 (25)	
Pathological T-status					0.069
pT1-2	20 (31)	37 (27)	6 (38)	7 (58)	
pT3-4	113 (69)	98 (73)	10 (62)	5 (42)	
Pathological N-status					0.901
pN0-1	77 (48)	64 (48)	8 (50)	5 (42)	
pN2	85 (52)	70 (52)	8 (50)	7 (58)	
Pathological stage					0.832
p-stage I–III	36 (22)	31 (23)	3 (19)	2 (17)	
p-stage IV	127 (78)	104 (77)	13 (81)	10 (83)	
Extracapsular spread					0.897
No	88 (54)	74 (55)	8 (50)	6 (50)	
Yes	75 (46)	61 (45)	8 (50)	6 (50)	
Level IV/V metastases					0.563
No	155 (95)	128 (95)	16 (100)	11 (92)	
Yes	8 (5)	7 (5)	0	1 (8)	
Treatment mode					0.652
Surgery plus RT	74 (45)	62 (46)	8 (50)	4 (33)	
Surgery plus CCRT	89 (55)	73 (54)	8 (50)	8 (67)	
Local recurrence					0.942
No	141 (86)	117 (87)	14 (87)	10 (83)	
Yes	22 (14)	18 (13)	2 (13)	2 (17)	
Neck recurrence					0.645
No	133 (82)	109 (81)	13 (81)	11 (92)	
Yes	30 (18)	26 (19)	3 (19)	1 (8)	
Distant metastases					0.064
No	128 (78)	110 (81)	9 (56)	9 (75)	
Yes	35 (22)	25 (19)	7 (44)	3 (25)	
Relapse					0.392
No	97 (59)	83 (61)	7 (44)	7 (58)	
Yes	66 (41)	52 (39)	9 (56)	5 (42)	
Secondary primary tumors					0.351
No	124 (76)	102 (76)	11 (69)	11 (92)	
Yes	39 (24)	33 (24)	5 (31)	1 (8)	
Death					0.132
No	78 (48)	67 (50)	4 (25)	7 (58)	
Yes	85 (52)	68 (50)	12 (75)	5 (42)	
Cause of death					0.316
Primary cancer	56 (34)	43 (32)	9 (56)	4 (33)	
Other cancers	17 (10)	16 (12)	1 (6)	0 (0)	
Noncancer causes	12 (7)	9 (7)	2 (13)	1 (8)	

OSCC: oral squamous cell carcinoma. HPV: human papillomavirus. *n:* number of patients. HPV (-): HPV-negative. T: primary tumor. N: regional lymph node. RT: radiotherapy. CCRT: concomitant chemoradiation therapy.

a All cases comprised HPV (-), solitary HPV-16, and solitary HPV-18 patients.

### Combining HPV-16 with Traditional Prognostic Factors in Advanced OSCC Patients

Compared with the HPV-16-negative patients using the univariate analysis, the advanced OSCC patients with solitary HPV-16 infections had a significantly higher rate of distant metastases (56% vs. 19%, P = 0.007) and markedly lower DSS and OS rates (DSS: 37% vs. 68%, P = 0.025; OS: 25% vs. 53%, P = 0.028). The local control and neck control status were similar in both groups (local control: 87% vs. 84%, P = 0.774; neck control: 77% vs. 82%, P = 0.726). The multivariate analyses of important risk factors, including solitary HPV-16 infection, pN2, level IV/V metastases, ECS, tumor depth ≥11 mm, and lymphatic invasion, are shown in Table 3. Solitary HPV-16 infection was a significant independent predictor of 5-year distant metastases (hazard ratio [HR], 3.4; 95% confidence interval [CI], 1.4–8.0; P = 0.005), DFS (HR, 2.1; 95% CI, 1.0–4.3; P = 0.037), DSS (HR, 2.8; 95% CI, 1.4–5.8; P = 0.006), and OS (HR, 2.3; 95% CI, 1.2–4.3; P = 0.010). Other independent risk factors for 5-year distant metastases included neck level IV/V metastases, ECS, and tumor depth ≥11 mm. Except for pN2, HPV-16 and the other risk factors could independently predict 5-year OS. HPV-16, pN2, and level IV/V metastases were independent predictors of 5-year DFS, whereas these risk factors and tumor depth ≥11 mm were important risk factors of 5-year DSS.

**Table 3:** Multivariate analyses of 5-year control and survival rates in advanced OSCC patients (*n* = 173).

Risk factors	Local control	Neck control	Distant metastasis	Disease-free survival	Disease-specific survival	Overall survival
	*P*, HR (95%CI)	*P*, HR (95%CI)	*P,* HR (95%CI)	*P*, HR (95%CI)	*P*, HR (95%CI)	*P*, HR (95%CI)
Solitary HPV-16 (*n* = 16 )	ns	ns	0.005, 3.4 (1.4–8.0)	0.037, 2.1 (1.0–4.3)	0.006, 2.8 (1.4–5.8)	0.010, 2.3 (1.2–4.3)
pN2 (*n* = 88)	ns	0.011, 2.9 (1.3–6.7)	ns	0.001, 2.5 (1.5–4.1)	<0.001, 3.2 (1.8–5.7)	ns
Level IV/V metastasis(*n = *8 )	ns	0.026, 3.4 (1.2–10.2)	<0.001, 5.2 (2.1–13.3)	0.024, 2.75 (1.1–6.5)	0.042, 2.5 (1.0–6.0)	0.034, 2.4 (1.1–5.6)
Extracapsular spread(*n* = 78)	ns	ns	<0.001, 4.7 (2.1–10.6)	ns	ns	0.001, 2.2 (1.4–3.5)
Tumor depth ≥11 mm(*n* = 103)	ns	ns	0.009, 3.1 (1.3–7.1)	ns	0.001, 2.8 (1.5–5.2)	0.012, 1.8 (1.1–2.8)
Lymphatic invasion(*n* = 13)	ns	ns	ns	ns	ns	0.049, 1.9 (1.0–3.7)

OSCC: oral squamous cell carcinoma. *n*: patient number. HR: hazard ratio. CI: confidence interval. HPV: human papillomavirus. ns, not significant. pN: pathological lymph node metastases.

Table 4 presents the demographic, clinical, pathological, and therapeutic characteristics of the 16 advanced OSCC patients who were infected with HPV-16. Eight of these patients underwent CCRT due to ECS (100%) and had significantly higher risks of distant metastases than those that underwent RT (75% vs. 13%, P = 0.041). Moreover, 75% of the 16 patients who were dead at the end of study had died of the following causes, by decreasing order of frequency: disease (56%), other cancers (6%), or other reasons (13%). Accordingly, the major impact of HPV-16 on 5-year survival was due to the failure of treatment at distant sites resulting in death; DSS in patients with distant metastases was significantly lower than that in patients without distant metastasis (22% vs. 100%, P = 0.003). Of note, none of the 16 patients had level IV/V metastases (Tables 1 and 3).

**Table 4:** Demographic, clinical, pathological, and therapeutic characteristics of the advanced OSCC patients with solitary HPV-16 infections.

No.	Sex	Age (y)	Oral habit	Site	p-Stage	ECS	Level IV/V metastases	Tumor depth (mm)	Adjuvant therapy	Interval between primary surgery and relapse (mo)				
										TR (mo)	NR (mo)	DM (mo)	Salvage	Outcomes (mo)
1	m	58	a, b, c	buccal	T3N1	-	-	1	RT					DOO, 2
2	m	58	a, b, c	gum	T4N2b	+	-	35	CCRT			7	–	DOD, 7
3	m	51	b, c	buccal	T4N1	-	-	25	RT	6			–	DOD, 8
4	m	25	a, b, c	gum	T4N2b	+	-	12	CCRT			7	–	DOD, 8
5	m	47	a, b, c	tongue	T4N1	-	-	13	RT	5			CCRT	DOD, 14
6	m	62	a, b, c	buccal	T4N0	-	-	50	RT					DOO, 15
7	m	73	a, c	tongue	T2N2b	+	-	6	CCRT		7	8	CCRT	DOD,15
8	m	69	b, c	buccal	T2N2b	+	-	22	CCRT			18	–	DOD, 19
9	m	31	a, b, c	gum	T2N2b	+	-	9	CCRT		7	18	CCRT	DOD, 24
10	m	47	b, c	buccal	T2N1	+	-	14	CCRT		15	16	–	DOD, 25
11	m	60	a, b, c	gum	T4N0	-	-	13	RT			21	–	DOD, 26
12	m	47	a, b, c	tongue	T4N0	-	-	12	RT					DOC, 37
13	m	69	a, b, c	gum	T4N2c	+	-	10	CCRT					NER, 61
14	m	45	a, b, c	mouth floor	T2N2b	+	-	2	CCRT					NER, 70
15	f	54	B	tongue	T2N2b	-	-	13	RT					NER, 79
16	m	47	a, b, c	tongue	T3N0	-	-	24	RT					NER, 90

OSCC, oral squamous cell carcinoma; HPV, human papillomavirus; m, male; f, female; a, alcohol drinking; b, betel nut chewing; c, cigarette smoking; p, pathological; ECS, extracapsular spread; mo: month; TR, tumor recurrence; NR, neck recurrence; DM, distant metastases; RT, radiotherapy; CCRT, concomitant chemoradiation; DOO, died of other reason; DOC, died of other cancer; DOD, died of disease; NER, no evidence of recurrence.

### Post Hoc Analyses for 5-year Distant Metastasis Rates in Advanced OSCC Patients

**Figure 3:**
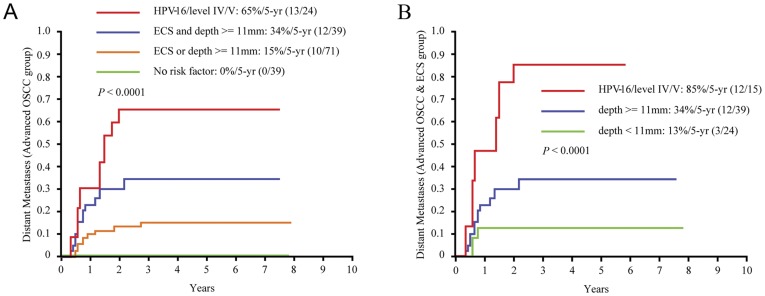
Five-year distant metastases in patients with advanced OSCC. A, distant metastases by newly classified subgroups in the advanced OSCC patients; B, distant metastases according to the new risk stratification in advanced OSCC patients with ECS.

Using level IV/V metastases, solitary HPV-16 infection, ECS, and tumor depth ≥11 mm as independent predictors, we created four subgroups (5-year distant metastases: 83%, 56%, 38%, and 30%, respectively): an “HPV-16 or level IV/V metastases” group (n = 24), a “both ECS and tumor depth ≥11 mm” group (n = 39), an “ECS or tumor depth ≥11 mm” group (n = 71), and a “no risk factor” group (n = 39). The 5-year distant metastatic rate was higher in the HPV-16 or level IV/V group than in the ECS and tumor depth ≥11 mm group (P = 0.075), the ECS or tumor depth ≥11 mm group (P<0.001), and the no risk factor group (P<0.001) (Fig. 3A).

Among the 173 advanced OSCC patients, 78 had ECS diseases; these ECS patients were divided in 3 subgroups based on the occurrence of 5-year distant metastases (event/*n*): the HPV-16 (6/8) or level IV/V (6/7) group, the tumor depth ≥11 mm group (12/39), and the tumor depth <11 mm group (3/24). The 5-year distant metastasis rates of the ECS patients were higher in the HPV-16 or level IV/V group than in the tumor depth ≥11 mm group (*P* = 0.004, HR = 3.2, 95% CI = 1.4–7.3) and the tumor depth <11 mm group (*P*<0.001, HR = 9.3, 95% CI = 2.6–33.4) (Fig. 3B).

## Discussion

The null hypothesis of the present study was rejected, that is, HPV infections did not decrease the risk of disease relapse and was not associated with a better survival among advanced OSCC patients undergoing radical surgery and radiation-based adjuvant therapy (Fig. 1 & Fig. 2). By contrast, advanced OSCC patients with a solitary HPV-16 infection were 3 times more likely to develop distant metastases and were 2–3 times more likely to die earlier (including those who died due to DFS, DSS, or OS) compared with HPV-negative patients (Table 3). Among the OSCC patients, including uncomplicated and advanced cases, the prevalence of HPV infection was 21.3%. The HPV-positive patients had similar rates of DFS and DSS, but a lower OS rate, compared with the HPV-negative patients (Fig. 1). Radical surgery seemed to be sufficiently effective for uncomplicated OSCC regardless of the HPV status, whereas radiation-based adjuvant therapy was unsatisfactory in treating HPV-16-positive advanced OSCC. In contrast to HPV-18 infection, HPV-16 infection had a negative impact on distant metastases, DFS, DSS, and OS despite immediately postoperative adjuvant therapy (Fig. 2).

Our findings are surprisingly different from the recent HPV-related outcome surveys in the field of head and neck cancers that have shown positive clinical impacts of HPV on DSS or OS [1]–[10], [13], [14], [24], [25]. Moreover, only a few studies have assessed failure patterns and time to relapse [1], [8], [9], especially with regard to cancers of the oral cavity [9]. This lack of attention to OSCC might be because some studies had relatively lower rates of detecting HPV (<10%), small samples, or included patients with cancers in different subsites (i.e., both the oral cavity and oropharynx) [3], [4], [10], [12]–[15], [24], [26]–[28]. For this reason, different treatment modalities were used (surgery vs. RT/CCRT), ultimately leading to different outcomes. Other possible explanations are that the enrolled patients came from different regions and were exposed to different carcinogens (e.g., betel quid chewing), different cultural norms (e.g., habitual oral sex behavior) and different genetic backgrounds (e.g., HLA typing) [29].

The evidence regarding the clinical impact of HPV in OSCC patients remains inconclusive [6], [12], [13], [28], [30]. HPV-seropositive heavy smokers or heavy drinkers are at a significantly higher risk of having OSCC than HPV-seronegative heavy smokers or drinkers [30]. Accordingly, it is reasonable that most of the HPV-positive OSCC patients smoke cigarettes and/or drink alcohol (Table 2). Based on Maxwell’s findings, cigarette smoking remarkably increases the risk of local recurrence and distant metastases among HPV-positive oropharyngeal cancer [31]. Smoking may induce genetic mutations, which facilitate the integration of HPV DNA into the host genome, and causes somatic gene errors. The sophisticated relationships among these oncogenic agents, including tobacco, smoking, and HPV, and tumor control are particularly difficult to clarify when most of the advanced OSCC patients within Taiwan have been exposed to tobacco and/or alcohol regardless of the HPV status. However, HPV-16 infection is an important, independent predictor of worse outcome among advanced OSCC patients, even those who underwent extensive operations and received adjuvant therapy. To our knowledge, longstanding betel quid chewing can damage the HPV-infected epithelia of the oral cavity and can potentially lead to a significant accumulation of chemicals, which may also influence the carcinogenic effect of HPV, and probably results in clinical and biological discriminations between HPV-positive OSCC and HPV-positive oropharyngeal cancer. Therefore, additional studies are needed to examine the significance of HPV infection in the presence or absence of betel nut chewing; this knowledge may be helpful to elucidate the genetic alterations and the molecular pathways that may underlie the observed survival differences.

HPV-18 infection is uncommon (<10% of all HPV infections) in oropharyngeal cancer [11] but is frequently found (32%, *n* = 12) in advanced OSCC. However, there was no difference in relapse or survival between patients with and without solitary HPV-18 infections. In this context, we further focused our study on HPV-16. Previous studies have reported that HPV-positive oropharyngeal cancers are associated with poorly differentiated histology, T1–T2 disease, N2–N3, and radiosensitivity [1]–[11]. By contrast, we observed that HPV-16-positive advanced OSCC cases have a similar status in terms of T-staging/N-staging and a remarkably higher incidence of distant metastases within two years of radical surgery (56%, 7/16) compared with HPV-negative cases. Even after adjuvant therapy, the advanced OSCC patients with solitary HPV-16 infection still had a relative higher risk of early distant metastases (Fig. 2A). We believe that the possible survival benefit of HPV-16 might be diminished by oral habits or reduced by surgery; however, early diagnosis and adequate radical surgery are still the most important measures in the control of OSCC tumors.

In addition to solitary HPV-16 infection, we further showed that level IV/V metastases, ECS, and tumor depth ≥11 mm are independent risk factors for 5-year distant metastases in advanced OSCC. Four subgroups of distant metastases were thus created (Fig. 3A). We previously demonstrated that OSCC patients with ECS have a higher potential for distant metastases than other groups of patients [32]. In this study, distant metastases were found in 6 of the 7 OSCC patients with level IV/V metastases. Among the HPV-16-positive advanced OSCC patients, six of 8 cases with ECS developed distant metastases (Table 3 and Fig. 3B). Accordingly, more intensive and specific treatments should be administered in OSCC patients with level IV/V metastases or HPV-16 with ECS, such as taxane-based chemotherapy regimens as an adjuvant strategy immediately following radical surgery; transitions to alternative, palliative treatments; biotherapy; and anti-angiogenesis strategies utilized during the postoperative recovery period [33], [34].

Several caveats of this study merit comment. First, a potential limitation of our report is the use of specific PCR assays for detecting the HPV L1 gene. Because PCR amplification of HPV DNA is a very sensitive technique, we ruled out the possibility of laboratory artifacts and the presence of environmental virions by performing all amplifications in duplicate and with the use of two different PCR assays. We acknowledge that reverse transcriptase-PCR assays for E6 and E7 transcripts may be more reliable for the detection of oncogenic HPV infections. Moreover, the lack of available tumor marker data (e.g., p16, p53, and epidermal growth factor receptor) does not allow us to draw any conclusion on the activity of viral oncogenes. To further characterize the possible mechanisms by which HPV-16 infections could be related to the risk of distant metastases and death, the measurement of E6 and E7 expression and HPV-associated biomarkers will be required in future studies.

In conclusion, HPV infection does not represent a favorable prognosticator in the OSCC patients who receive radical surgery regardless of radiation-based adjuvant therapy. Notably, the advanced OSCC patients with solitary HPV-16 infection require priority adjuvant treatment and follow-up due to increased risk of early distant metastases and death. Moreover, in particular, patients with level IV/V metastases or HPV-16 infection with ECS require a more intensive therapeutic protocol. Our findings suggest that different types of HPV infections present distinct clinical and biological challenges among advanced OSCC patients, and there is at least one such HPV-16 associated with unfavorable outcomes in individuals who have received conventional adjuvant treatment. The significance of HPV-16 infection should be further studied in future translational and clinical research.
